# TTC7A deficiency: A retrospective international study on treatment and outcomes from the Inborn Errors Working Party of EBMT

**DOI:** 10.70962/jhi.20250271

**Published:** 2026-07-08

**Authors:** Giulia Prunotto, Marie Parreillet, Bénédicte Neven, Despina Moshous, Genevieve de Saint Basile, Caroline Lindemans, Timothy Ronan Leahy, Elie Haddad, Herbert Pichler, Joao Farela Neves, Mary Slatter, Nasrine Radwan, Carsten Speckmann, Christos Tzivinikos, Edward J. Hoffenberg, Chee Y. Ooi, Alberto Pinzon-Charry, Looi C. Ee, Silvia Ricci, Michael H. Albert, Jonathan O’Donnell, Austen Worth, Holm H. Uhlig, Aleixo Muise, Giovanna Lucchini

**Affiliations:** 1 Fondazione IRCCS San Gerardo dei Tintori, Monza, Italy; 2 Pediatric Immuno-Hematology and Rheumatology Unit, Necker Hospital, Assistance Publique Hopitaux De Paris, Paris, France; 3 Institut des Maladies Génétiques (Imagine) Institute, University Paris Cité, Paris, France; 4 Princess Máxima Center for Pediatric Oncology, Utrecht, Netherlands; 5 Department of Pediatrics, University Medical Center Utrecht, Utrecht, Netherlands; 6 Children’s Health Ireland at Crumlin, Trinity College, Dublin and University of Dublin, Dublin, Ireland; 7 Department of Pediatrics, CHU Sainte-Justine Azrieli Research Center, University of Montreal, Montreal, Canada; 8 Department of Microbiology, Immunology and Infectious Diseases, CHU Sainte-Justine Azrieli Research Center, University of Montreal, Montreal, Canada; 9 Department of Pediatrics and Adolescent Medicine, St. Anna Children’s Hospital, Austria and St. Anna Children’s Cancer Research Institute, Medical University of Vienna, Vienna, Austria; 10 Hospital Dona Estefânia, Centro Hospitalar Universitário de Lisboa Central, Lisbon, Portugal; 11 Great Northern Children’s Hospital, Newcastle Upon Tyne, UK; 12 Children’s Hospital, Ain Shams University, Cairo, Egypt; 13 Institute for Immunodeficiency, Center for Chronic Immunodeficiency, Faculty of Medicine, Medical Center-University of Freiburg, University of Freiburg, Freiburg, Germany; 14 Department of Pediatric Hematology, Oncology and Stem Cell Transplantation, Children’s Hospital, Faculty of Medicine, Medical Center – University of Freiburg, University of Freiburg, Germany; 15 Al Jalila Children’s Specialty Hospital, Dubai, UAE; 16 Children’s Hospital, Aurora, CO, USA; 17 Discipline of Paediatrics & Child Health, School of Clinical Medicine, UNSW Medicine & Health, University of New South Wales, Sydney, Australia; 18 Queensland Children’s Hospital, University of Queensland and Griffith University, Brisbane, Australia; 19 Meyer Children’s Hospital, IRCCS, Florence, Italy; 20 Department of Pediatrics, Dr. von Hauner Children’s Hospital, University Hospital, LMU Munich, Munich, Germany; 21 SickKids Inflammatory Bowel Disease Center and Cell Biology Program, Research Institute, Hospital for Sick Children, Toronto, Canada; 22 Great Ormond Street Hospital NHS Foundation Trust, London, UK; 23 Department of Paediatrics, Centre for Human Genetics, Biomedical Research Centre, University of Oxford, Oxford, UK

## Abstract

Tetratricopeptide repeat domain 7A (TTC7A) deficiency is a primary immunodeficiency that occurs due to mutations in the *TTC7A* gene. It causes intestinal disease and a poorly characterized immunodeficiency, with poor long-term survival. We describe the clinical and immunological characteristics, management, and outcomes of an international cohort of patients with genetically confirmed TTC7A deficiency. Data from 61 patients from 13 countries were retrospectively analyzed. Overall survival was 64% (median follow-up 4.2 years), significantly higher in the inflammatory bowel disease group than in the hereditary multiple intestinal atresia group (80 vs. 48%, P < 0.05). Infections represent the leading cause of death. Allogeneic stem cell transplantation was performed in 16 patients to correct the immunodeficiency, but it was associated with significant transplant-related mortality (44%). Solid organ transplantation of the small bowel remains exceptionally rare (two patients). Malignancy/autoimmune disorders developed in 4 (6.5%) and 17 (28%) patients, respectively. TTC7A deficiency remains difficult to treat, and prognosis is dismal for affected patients. Further understanding of the disease mechanisms and development of innovative treatment approaches are required.

## Teaser

Prunotto et al. describe clinical presentation, immunological status, treatment, and long-term outcomes of a large cohort of pediatric and adult patients affected by an extremely complex and rare immunodeficiency known as TTC7A deficiency for which no clear therapeutic pathway is currently available.

## Introduction

Tetratricopeptide repeat domain 7A (TTC7A) is a protein that regulates epithelial polarity and survival through its interaction with the phosphoinositide-generating enzyme phosphatidylinositol 4-kinase IIIα. Defects in TTC7A function lead to loss of apicobasal polarity, disruption of cell adhesion, and increased apoptosis in the gastrointestinal epithelium (1, 2, 3).

TTC7A deficiency was first reported in 2013, and since then, 79 TTC7A variants have been described (4, 5, 6). Complete loss of function due to truncating mutations is predicted to result in more severe phenotypes (1, 7, 8).

The literature documents a range of phenotypic variations among patients with TTC7A deficiency. Gastrointestinal manifestations may present as multiple intestinal atresia with varying degrees of enteropathy, or as enteropathy without atresia. Both of these phenotypes can be associated with combined immunodeficiency of variable severity.

The hereditary multiple intestinal atresia (HMIA) phenotype presents as discontinuity of the intestinal lumen caused by obstructions anywhere from the pylorus to the rectum, while the enteropathy phenotype presents with inflammatory bowel disease (IBD)–like symptoms (7, 9). In some cases, progressive liver dysfunction, including fibrotic and cholestatic changes, has been reported in association with the intestinal phenotype (10).

Global mortality among TTC7A-deficient patients is high, reported at 65.8%, with a mean age at death of 11.8 mo. Mortality is significantly higher in the HMIA group. Genotype–phenotype correlations indicate that the HMIA phenotype is strongly associated with double nonsense variants (77.8%), whereas the IBD phenotype is predominantly associated with double missense variants (73.7%; P < 0.001) (5, 7). Patients with the enteropathy phenotype may also develop autoimmune manifestations in the second and third decades of life (1); however, data on neoplastic risk or secondary organ involvement are lacking.

Although gastrointestinal features are relatively well described, immunodeficiency-related symptoms are less well understood. Immune dysregulation is observed in over 75% of patients, ranging from autoimmune manifestations to common variable immune deficiency or severe combined immunodeficiency (SCID)–like presentations (7, 10). Postmortem examination of the thymus in affected patients reveals severe atrophy, loss of corticomedullary demarcation, and marked lymphoid depletion, though the clinical significance of these alterations and their impact on treatment choice remains unclear (8).

Currently, no standard of care exists for TTC7A deficiency. Surgical resection is often performed in patients with intestinal atresia at a young age, but this does not prevent recurrence. Enteropathy is typically refractory to immunosuppressive (IS) therapies, steroids, and biologics. Leflunomide has demonstrated potential in vitro models by reducing epithelial apoptosis, though in vivo data are limited (1, 11, 12). Hematopoietic stem cell transplantation (HSCT) may correct the immunodeficiency and improve survival in patients with immunodeficiency, but it does not appear to address intestinal epithelial defects and carries a high risk of transplant-related mortality (1, 13). Intestinal or liver transplantation has been attempted in a limited subset of patients to correct intestinal atresia, but the complexity of the procedure limits its feasibility.

Since TTC7A deficiency is a disorder with a poor prognosis and most information is derived from smaller case series, additional data are needed to improve understanding, management, and outcomes. In this study, we retrospectively collected data from the largest reported, international cohort of patients with genetically confirmed TTC7A deficiency to describe clinical and immunological characteristics, management, and outcomes.

## Results

### Patient characteristics

Patient characteristics are summarized in Table 1. Data were reported to our study by immunologists (*n* = 44), gastroenterologists (*n* = 6), or stem cell transplant specialists (*n* = 11). Most patients were diagnosed at birth or shortly thereafter, with a median age at genetic diagnosis of 1.24 years (range 0–51). Genetic findings are detailed in Table 2; six patients belonged to a large family sharing the same mutation, as previously reported (4, 8). 31 patients had an HMIA phenotype, and 30 had IBD.

**Table 1.** Patient characteristics at diagnosis

​	HMIA (*n* = 31)	IBD (*n* = 30)
Age at symptom presentation	Prenatal: 11 (36%)	Prenatal: 0
	At birth: 15 (48%)	At birth: 7 (23%)
	After birth: 5 (16%)	After birth: 22 (77%) (missing data 1)
Median age at genetic diagnosis (years)	0.58 (0–29)	2.91 (0–51)
Gender	M: 14 (45%)	M: 18 (60%)
	F: 17 (55%)	F: 12 (40%)
Liver derangement at diagnosis	6 (20%) (missing data 2)	6 (20%) (missing data 3)
Surgery at presentation	28 (90%)	3 (10%)
PN	30/31 (97%)	15/30 (50%)
Median age at PN start (years)	0 (0–0.25)	0.28 (0–1.30)
Indication to PN	Partial enteral tolerance: 2 (6%)	Partial enteral tolerance: 12 (80%)
	No enteral tolerance: 27 (90%)	No enteral tolerance: 3 (20%)
	Surgery/transplant support: 1 (4%)	Surgery/transplant support: 0

M, male; F, female.

**Table 2.** Genetic data

Mutation		Type of mutation	Predicted residual protein function	Phenotype
c.211G>A (p.Glu71Lys)	Homozygous	Missense	Partial loss (hypomorphic)	IBD
c.1008C>G (p.Y336X)+c.1479delG (p.L493fsX13)	Compound heterozygous	Nonsense+	None (loss of function)	HMIA
		frameshift		
c.975G>A (p.R325Q)	Homozygous	Missense	Partial loss (hypomorphic)	IBD
c.313_316delTATC+c.1479delG (p.L493fsX13)	Compound heterozygous	Frameshift/premature stop + splice site	None (loss of function)	IBD
c.2356-1G>A	Homozygous	Canonical splice acceptor	None (loss of function)	HMIA
c.1373G>A (p.Cys458Tyr)+c.1433T>C (p.Leu478Pro)	Compound heterozygous	Missense + missense	Partial loss (hypomorphic)	IBD
c.1433T>C (p.L478P)+c.2495C>T (p.A832V)	Compound heterozygous	Missense + missense	Partial loss (hypomorphic)	IBD
p.Ser539Leu+p.Ala839Thr	Compound heterozygous	Missense + missense	Partial loss (hypomorphic)	IBD
p.Gly173Val+p.Leu452Pro	Compound heterozygous	Missense + missense	Partial loss (hypomorphic)	IBD
c.295A>G (p.M99V)	Homozygous	Missense	Partial loss (hypomorphic)	HMIA
c.1190delCA	Homozygous	Frameshift	None (loss of function)	HMIA
c.192delT (p.Phe64Leufs*15)	Homozygous	Frameshift	None (loss of function)	HMIA
c.1073G>A (p.Arg358Gln)	Homozygous	Missense	Partial loss (hypomorphic)	HMIA
c.518G>A (p.Gly173Asp)+	Compound heterozygous	Missense+	Partial loss (hypomorphic)+	HMIA
c.100_1001+2delAAGT+		Frameshift/splice+	None (loss of function)+	
c.2170C>A (p.Gln724Lys)		Missense	Partial loss (hypomorphic)	
c.1001+3_1001+6del+	Compound heterozygous	Splice region+	Likely loss of function	HMIA
c.2470dup+		Frameshift+	None (loss of function)	
c.1364C>A (p.Ala455Asp)		Missense	Partial loss (hypomorphic)	
c.517+1G>C+c.2225-2A>G	Compound heterozygous	Splice site (donor) + splice site (acceptor)	None (loss of function)	HMIA
c.728C>A (p.Ala243Asp)+	Compound heterozygous	Missense	Partial loss (hypomorphic)	HMIA
c.1480T>C (p.Gly494Cys)				
c.1027G>A (p.Gly343Ser)+	Compound heterozygous	Missense	Partial loss (hypomorphic)	IBD
c.2405T>C (p.Ile802Thr)				
c.765_1065del (p.N256Qfs*7)	Homozygous	Frameshift	None (loss of function)	HMIA
c.185-517del + c.185-348del	Compound heterozygous	Intronic deletion	Partial loss (hypomorphic)	HMIA
c.1709A>G (p.His570Arg)	Homozygous	Missense	Partial loss (hypomorphic)	HMIA
c.189C>G (p.D63E)+	Compound heterozygous	Missense+	Partial loss (hypomorphic)+	HMIA
c.412C>T (p.R138X)		Nonsense	None (loss of function)	
c.1636C>T (p.Arg546Trp)	Homozygous	Missense	Partial loss (hypomorphic)	HMIA
c.1569-2A>G+	Compound heterozygous	Spice site (acceptor)	None (loss of function)+	HMIA
c.1571C>T (p.A524V)		Missense	Partial loss (hypomorphic)	
p.Glu191fs+	Compound heterozygous	Frameshift+	None (loss of function)+	HMIA
p.Ile854Phe		Missense	Partial loss (hypomorphic)	
c.900C>G (p.Tyr300*)+	Compound heterozygous	Nonsense+	None (loss of function)+	HMIA
c.1213C>T (p.Arg405Cys)		Missense	Partial loss (hypomorphic)	
p.Glu71Lys+	Compound heterozygous	Missense+	Partial loss (hypomorphic)+	HMIA
p.Glu96		Nonsense	None (loss of function)	
p.Gly45_Ala55del	Homozygous	In-frame deletion	Partial loss of function	HMIA

List of the identified mutations in our population of patients and correlation with the presented clinical phenotype. IBD, inflammatory bowel disease; HMIA, hereditary multiple intestinal atresia.

Liver biochemistry derangement at presentation was observed in 20% of patients in both phenotypes. As expected, the need for surgery at presentation was significantly higher in HMIA compared to IBD patients, with 28 (90%) and 3 (10%), respectively (P < 0.05). Almost all surgeries in patients with HMIA involved multiple resections of atretic segments of the gut and the creation of a stoma at various anatomical levels, ranging from the duodenum to the colon, depending on the extent of the strictures. Pyloric or antral web resections were reported in three cases, pyloroplasty in two, anal dilatation in one, and decompression for a meconium plug in a single case. In the IBD cohort, only 3 of 30 patients (10%) underwent surgery: one underwent omphalocele repair, a second had an ileostomy created, and surgical details for the third patient were not reported.

In the HMIA group, parenteral nutrition (PN) was needed in the vast majority of patients (*n* = 30, 97%), starting at birth in most cases. In the IBD cohort, PN was required in 15 patients (50%) at a median age of 3.36 mo. Indications for PN are detailed in Table 1.

### Immunology at diagnosis

Newborn screening with TREC analysis was largely unavailable at these patients’ birth centers; TREC results from Guthrie cards were available in eight patients and were abnormal in one (HMIA). HMIA patients exhibited more severe immune abnormalities, with T cell lymphopenia in 92 vs. 66% in IBD. At diagnosis, CD3 cell counts were below the age reference range in 23 (74%) of HMIA and in 13 (43%) of IBD patients, and CD19 cell counts were lower than normal in 8 (26%) of HMIA and 5 (17%) of IBD patients. Immunoglobulin replacement at diagnosis was more frequently required in HMIA (71 vs. 43%, P < 0.05), consistent with a trend toward lower B cell counts. Natural killer cell numbers were comparable. Cytopenias were rare (one neutropenia, one thrombocytopenia in HMIA).

### Infections

There was no significant difference in the incidence of opportunistic or deep-seated infections between the two groups at diagnosis (Table 3). The most frequently reported were CMV-related (in six patients) followed by Gram-negative bacterial infections (one meningoencephalitis, one *Serratia marcescens* peritonitis, one *Escherichia coli* pyelonephritis, one *Pseudomonas* species sepsis). A single case of aspergillosis was reported at diagnosis.

**Table 3.** Infections and management at diagnosis

​	HMIA (*n* = 31)	IBD (*n* = 30)	P value
Infections at dx	4 (13%)	9 (30%) (2 missing)	P = ns
Prophylaxis	Pneumocystis: 11 (35%)	Pneumocystis: 15 (60%)	P = ns
	Pneumocystis + antifungal: 11 (35%)	Pneumocystis + antifungal: 4 (16%)	
	Antifungal: 2 (7%)	Antifungal: 0	
	No: 7 (23%)	No: 6 (24%) (missing data 5)	
Ig replacement at dx	22 (85%) (missing data 5)	13 (43%) (missing data 1)	P < 0.05
Admission to ICU	10 (32%)	2 (6%)	P < 0.05

Ig, immunoglobulin; dx, diagnosis; ICU, intensive care unit.

70% of HMIA and 87% of IBD patients experienced an infection over time. More precisely, >90% of patients undergoing HSCT and between 70 and 80% of the nontransplanted ones developed infections during their clinical follow-up. At the time of data collection, 68 viral events in 33 patients off immunosuppression were reported in our cohort. Most of them were due to CMV reactivations (CMV disease in two cases) and pulmonary infections. One patient had severe VZV-related complications. 53 bacterial events were reported in 28 patients off immunosuppression, including 19 episodes of Gram-positive and Gram-negative septic events, highlighting the complex interplay between possible bacterial translocation from the gut and central line infections. *Pneumocystis jirovecii* pneumonia was reported in one subject.

Admission to intensive care units due to the need for ventilation or inotropic support was significantly higher in the HMIA than in the IBD group (32 vs. 6%, respectively, P < 0.05).

Overall, infection was deemed to be the cause of death in 50% of both HMIA and IBD patients, highlighting again the fragility of this population.

### Treatment

In the overall population, 45 of 61 (74%) needed PN, mainly in the HMIA group, as shown in Table 1. The median duration of PN was 2 years (0.05–17).

IS treatment was offered to treat gut-related symptoms in 32% of the whole population (8 [26%] HMIA and 11 [38%] IBD) (Table 4). The most common first-line IS agents were steroids or infliximab. Three patients received leflunomide as first-line treatment (one HMIA and two IBD), with variable response. Response to IS was limited in the cohort with a trend toward higher response rate in the IBD group (50 vs. 38%). Response was evaluated based on clinical readouts; no response was documented histologically. All responding patients showed a reduction of inflammatory gut symptoms; in a single IBD case, response to IS implied improved enteral feeding.

**Table 4.** IS treatment, description of lines of treatment used in patients by phenotype (HMIA vs. IBD)

HMIA					
First line of IS	Response	Second line of IS	Response	Third line of IS	Response
Steroid + CNI	NR	​	​	​	​
Steroid	NR	Mepolizumab	NR	Dupilumab + leflunomide	SD
Steroid	PR	Steroid + CNI	PR	Steroid + sirolimus	SD
Leflunomide	NR	​	​	​	​
Steroid + infliximab	PR	​	​	​	​
Steroid + infliximab	NR	​	​	​	​
Steroid	NR	​	​	​	​
Sirolimus + leflunomide	PR	​	​	​	​

IBD					
First line of IS	Response	Second line of IS	Response	Third line of IS	Response
Infliximab	GR	​	​	​	​
Leflunomide	PR	Infliximab	NR	Adalimumab	NR
Steroid	GR	​	​	​	​
Azathioprine	GR	​	​	​	​
Leflunomide	GR	​	​	​	​
Steroid + CNI	NR	​	​	​	​
Infliximab	NR	Sirolimus	NR	​	​
Steroid	NE	​	​	​	​
Infliximab	NR	​	​	​	​
Infliximab	NR	Sirolimus	NR	​	​

Investigators could define response to IS treatment by choosing between the following statements: clinical improvement in IBD-like symptoms, clinical improvement in stricturing event frequency or severity, improved enteral feeding tolerance, histopathology documented improvement. All responders were described as clinical improvement in IBD-like symptoms; in a single patient with the IBD phenotype, the response also implied improved enteral tolerance. NR, nonresponse; PR, partial response; GR, good response; NE, not evaluable; SD, stable disease; CNI, calcineurin inhibitor.

16 patients underwent HSCT (10 HMIA and 6 IBD) with the primary aim of correcting their immunodeficiency. Table 5 shows HSCT characteristics. Patients tended to be transplanted early in life, with a median age at HSCT of 1.16 years (0.3–12.0). The majority of patients were transplanted from a matched donor (related 29%, unrelated 42%). Both reduced-intensity conditioning (RIC) and myeloablative conditioning (MAC) were used (14). All patients achieved neutrophil engraftment and stable donor chimerism. 25% of the patients presented mixed chimerism at the last follow-up, all ≥50% donor in whole blood. At a median follow-up of 4.5 years, six of seven patients still alive post-HSCT had CD3 counts above 1 × 10^−9^/liters. There was an incidence of 50% of acute graft-versus-host disease (aGVHD), but no cases of chronic graft-versus-host disease (cGVHD) were reported. 9 of the 16 (56%) patients who underwent HSCT died, 7 of whom died from transplant-related complications (most commonly infections), giving a transplant-related mortality of 44%. RIC vs. MAC regimens did not offer a survival advantage in this cohort of patients (57% for RIC vs. 37.5% for MAC, P = 0.6).

**Table 5.** HSCT characteristics

HSCT characteristics (*N* = 16 patients, 10 HMIA and 6 IBD)	
Median age at HSCT (years)	1.16 (0.3–12.0)
Donor	MRD: 4 (29%)
	MUD: 6 (42%)
	MMUD: 4 (29%) (missing data 2)
Conditioning	RIC: 7 (47%)
	MAC: 8 (53%) (missing data 1)
aGVHD	8 (50%)
cGVHD	0 (0%)
Rejection	1 (6%)
Outcome	Alive at the last follow-up = 7 (44%)
​	Dead = 9 (56%)
Median follow-up	3.76 years

MRD, matched related donor; MUD, matched unrelated donor; MMUD, mismatched unrelated donor; RIC, reduced-intensity conditioning; MAC, myeloablative conditioning; aGVHD, acute graft-versus-host disease; cGVHD, chronic graft-versus-host disease.

Only two patients in the cohort received a solid organ transplantation (SOT), both with the HMIA phenotype. The two SOT procedures were a small bowel plus liver transplant and a small bowel transplant, both performed after a previous HSCT. Both patients died, in one case due to a secondary myelodysplastic syndrome 2 years after SOT and in one case from multi-organ failure after SOT.

### Survival and outcomes

The median follow-up from diagnosis for the entire cohort was 4.2 years (0–9.43).

**Figure 1.**
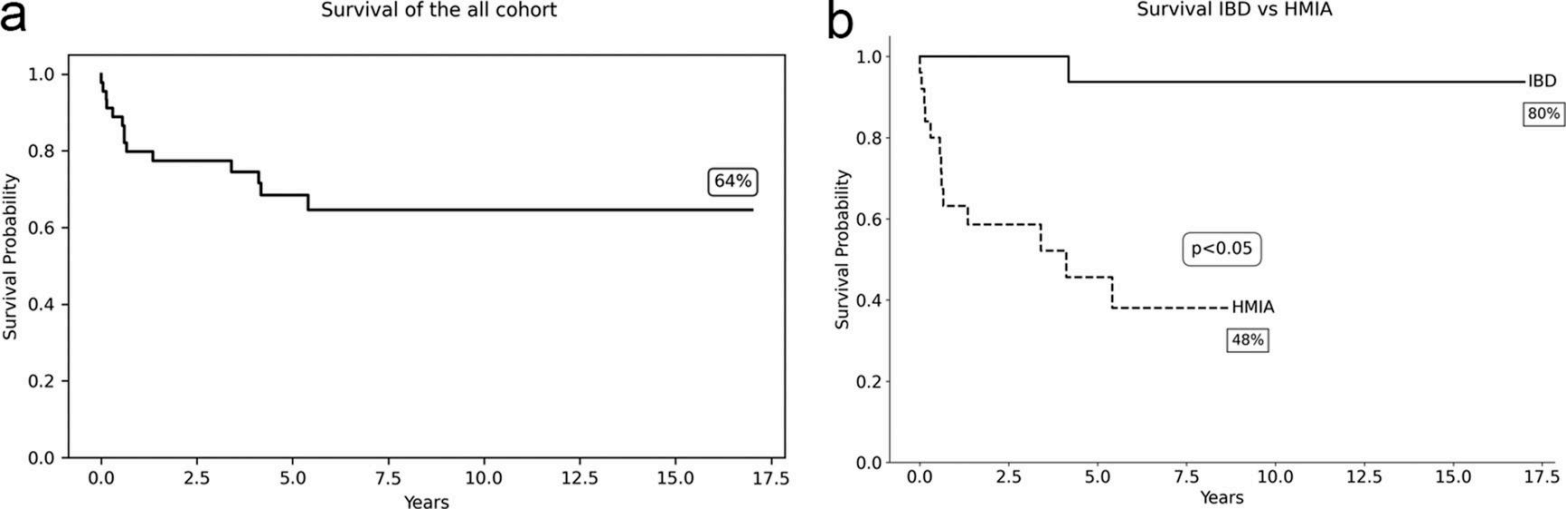
**Outcome. (a and b)** Overall survival for the whole cohort of patients (a) and for patients presenting with IBD vs. HMIA phenotype (b).

The survival curves are shown in Fig. 1. The survival of the whole cohort was 64% at the last follow-up. Overall survival was significantly higher in the IBD than in the HMIA group (80 vs. 48%, P < 0.05). The main causes of death were infections for both groups. Disease progression represented a significant cause of death in HMIA patients (25%) (Table 6). Autoimmune manifestations occurred in 28% of patients (12/30 in IBD, 5/31 in HMIA), most commonly cytopenia, thyroiditis, and alopecia.

**Table 6.** Causes of death

​	HMIA (16 patients)	IBD (6 patients)
Infections	8 (50%)	3 (50%)
Malignancy	1 (6%)	1 (16.6%)
TRM	3 (19%)	1 (16.6%)
Immune dysregulation	0	1 (16.6%)
Disease progression	4 (25%)	0

TRM, transplant-related mortality.

There were four cases of malignancies (three HMIA and one IBD): myelodysplastic syndrome, carcinoma of the stomach, lymphoblastic lymphoma (after HSCT), and a Langerhans cell histiocytosis (pre-HSCT).

Survival of IBD and HMIA patients according to transplant status is presented in Table 7.

**Table 7.** Survival of patients, HSCT vs. no HSCT

​	HMIA (*n* = 31)		P value	IBD (*n* = 30)		P value
	HSCT (*n* = 10, 32%)	No HSCT (*n* = 21, 68%)		HSCT (*n* = 6, 20%)	No HSCT (*n* = 24, 80%)	
Alive	4 (40%)	11 (52%)	P = 0.5	3 (50%)	21 (87.5%)	P = 0.04
Deceased	6 (60%)	10 (48%)		3 (50%)	3 (12.5%)	

Among patients with the HMIA phenotype, 15 survive long term: 4 following HSCT and 11 without HSCT.

Among the four HSCT survivors, none developed cGVHD, chronic lung disease, or malignancy. Three patients exhibit full donor chimerism, while one has 50% donor chimerism in the CD3 lineage. The three patients with full donor chimerism achieved adequate immune reconstitution with CD3 counts >1 × 10^9^/liter, although all remain on immunoglobulin replacement therapy. Two of the four patients developed autoimmune manifestations (thyroiditis and autoimmune thrombocytopenia), and two have progressive liver damage. None have discontinued PN, as they remain unable to tolerate enteral feeding.

Among the 11 long-term survivors without HSCT, 1 patient developed autoimmune thrombocytopenia, 2 have progressive liver damage, and 1 has chronic lung disease of unclear origin. 9 of 11 patients receive regular IVIG supplementation, and 6 and 11 have T cell counts below 0.3 × 10^9^/liter. All patients who required standard PN at diagnosis remain PN-dependent, with no tolerance to full enteral feeding to date.

Among patients with the IBD phenotype, 24 survive long term: 3 after HSCT and 21 without HSCT.

The three transplanted patients have no cGVHD, no lung or liver toxicity, and no malignancies. One patient developed autoimmune hemolytic anemia requiring ongoing IS therapy. All three show excellent immune reconstitution, with T cell counts exceeding 3 × 10^9^/liter, and two of three are no longer receiving immunoglobulin replacement. All patients are off PN, including two who required PN prior to HSCT.

**Figure 2.**
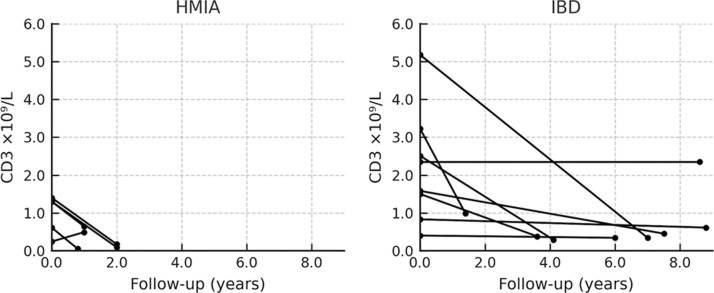
**CD3 cell count over time for patients divided by phenotype (HMIA vs. IBD) who were not on immunosuppression and had not received HSCT at their latest follow-up.** Each line represents a single patient.

Among the 21 long-term survivors without HSCT, 9 developed autoimmune manifestations and 4 are currently receiving IS therapy. 17 of 21 patients remain on immunoglobulin replacement therapy, and all but 1 maintain adequate T cell numbers, although a gradual decline over time is observed (Fig. 2). Notably, among the nine patients who required PN early in life, only one remains PN-dependent, while the others have successfully transitioned to full enteral feeding.

### Long-term disease status

39 of 61 patients are long-term survivors in our cohort (24 IBD and 15 HMIA) with a median follow-up of 5.47 years and a median age at the last follow-up of 7.5 years (0.5–56). Of these, 7 of 39 (18%) had received an HSCT. 24 surviving patients needed PN at some point. Of those, at the last follow-up, 16 (67%) are still PN-dependent due to partial/total enteral nutrition (14 HMIA and 2 IBD), while 8 (33%) were able to stop it. 30 patients are still on IVIG replacement, and 8 are on IS. We selected patients from both the HMIA and the IBD group who were not on immunosuppression and had not received HSCT at the last available follow-up. As shown in Fig. 2, in the IBD cohort, four of the seven patients shown demonstrate a clear decline in CD3 counts over time, whereas three appear to maintain relatively stable but low CD3 levels longitudinally. Patients with the HMIA phenotype are represented by very small numbers in this analysis, as most either died or underwent HSCT. However, the few patients depicted show a clear trend toward a decline in CD3 counts below the normal threshold within a relatively short time frame. The treating teams declared that 18% of the patients were potential candidates for further transplant procedures (either SOT or HSCT).

## Discussion

Our study represents the largest cohort of patients with genetically confirmed TTC7A deficiency described with dedicated information on immune status and treatment during the considered time frame. Some of the patients we included in this study had previously been reported, but not with their immunological details (8).

Consistent with previous data, we confirmed that there are significant differences between the HMIA and the IBD phenotype with regard to early surgical intervention and feeding tolerance. In patients with the IBD phenotype, 10 of 15 patients were able to stop PN at their latest follow-up, at median age of 1.5 years (0.4–3.2), confirming previous data from the French Canadian cohort (8) with regard to improvement of the intestinal phenotype for the IBD subset of patients over time. On the contrary, only 2 of 30 patients with PN dependence in the HMIA group were able to become PN-independent at the latest follow-up, irrespective of the treatment received.

Our data confirm prior observations that prenatal diagnosis is possible in severe HMIA phenotypes with features of gut atresia detected in prenatal scans (5).

Therefore, a diagnosis of TTC7A should be strongly considered in the prenatal and perinatal setting in patients with intestinal atresia, especially if associated with significantly reduced T cell counts. Liver derangement is not comprehensively described in TTC7A deficiency, but it was a relevant component of initial diagnosis for 20% of our patients, regardless of their phenotype. Histopathological observations are limited to patients who underwent SOT and highlight generic PN-related changes, which are not specific to the disease (9). Whether liver derangement at diagnosis simply reflects the severity of the phenotype, given the multifactorial causes of liver enzyme derangement, or whether this may be intrinsically related to genotype—or both—remains to be determined.

Our immunological data show that most TTC7A patients have decreased T cell numbers for age, some with a CID/SCID-like immunophenotype; this is specifically true for patients with HMIA. Our cohort is burdened by significant infections, and some of them are confirmed to be opportunistic (CMV, *Pneumocystis*, and *Aspergillus*), thus underlining the importance of long-term supportive multi-agent anti-infective prophylaxis. We note, however, that the level of severe T cell lymphopenia detected in the HMIA patients is not associated with life-threatening opportunistic infections in the first year of life, as one might expect. Among the patients with newborn testing for TRECs available, six had severe T-cell lymphopenia, but only one had low TRECs. This might suggest that thymic function is preserved early on and that loss through enteropathy might instead contribute to lymphocytopenia (and hypogammaglobulinemia) in the early phase of life. Data on long-term immunological evolution are limited in our cohort, but those available tend to show a progressive worsening of CD3 levels, which tend to be below the norm compared with the general population. Regarding transplanted patients, our data confirm that HSCT corrects the lymphopenia in the majority of patients as reported in the literature (13).

The majority of surviving patients remain on immunoglobulin replacement at the last follow-up, and importantly, a significant proportion of them develop autoimmune and/or malignant complications. This points toward an evolving immune deficiency/dysregulation as previously suggested by Lemoine and colleagues (2). Future studies should focus on functional studies of T and B cells to further elucidate the mechanism of immune imbalance and hopefully guide the clinical management of these patients. Patients who underwent HSCT seemed to be able to tolerate the acute transplant phase despite their young age and severe baseline conditions. However, mortality remained extremely high (56% in our cohort) particularly due to infectious comorbidities. Only 4 of 16 of HSCT patients became PN-independent at the last follow-up (3/6 IBD and 1/10 HMIA), suggesting that HSCT does not correct the gastrointestinal manifestations in HMIA, as previously reported (13). A higher percentage of patients receiving HSCT for the IBD phenotype seem to be able to stop PN after HSCT, but these data are difficult to interpret, as previous literature suggests that the IBD phenotype can become milder over the course of life in some subgroups of patients (2). The experience with SOT is too limited to draw definitive conclusions, but appropriately sized organ availability remains a major limiting factor, especially for younger patients. The combination of HSCT and SOT would theoretically address the complex nature of the disease, but remains very complex given the difficulty of immunosuppression, managing multisystem comorbidities and complications, as well as availability of these highly specialized procedures. Moreover, it showed fatal toxicity in the two patients of our cohort who have been treated with this approach.

In summary, there is no standard management to be recommended for TTC7A patients. Our data confirm a limited response to IS treatments, thus supporting the ongoing research of new molecules directed against specific cellular pathways, such as leflunomide or RhoA kinase inhibitors. HSCT should only be performed if a significant benefit from correction of the immunodeficiency can be expected. Supportive therapy remains the main pillar of care for children with TTC7A deficiency and consists of both extensive anti-infective prophylaxis and PN.

Future collaborative prospective studies could allow us to follow the evolution over time of these patients, both transplanted and nontransplanted, to better understand in particular the immunological aspects and identify possible alternative therapeutic strategies.

This study has several limitations. Firstly, given the rare nature of this condition, this was a retrospective multicenter study, with outcomes reported from sites around the world based on available data in each patient’s medical record. This may introduce recall bias, as well as reporting bias and center bias, into our study. Our variables of interest, such as liver derangement, infections, and need for PN, are all subject to multiple confounders.

This study reports on the largest cohort of TTC7A-deficient patients with granular immunological data. Our longitudinal study highlights profound T cell lymphopenia, a high burden of infections, and evolving immune dysregulation, particularly in HMIA patients. Conventional therapies, including immunosuppressants and HSCT, have limited efficacy for gastrointestinal manifestations, and supportive care remains central. These findings underscore the need for early diagnosis, comprehensive immunological monitoring, and the development of targeted therapies. Prospective, collaborative studies are essential to improve long-term outcomes and guide management strategies for this complex disorder.

## Materials and methods

This international, multicenter, retrospective study identified patients with a diagnosis of TTC7A deficiency due to biallelic TTC7A mutations identified using a CLIA-certified clinical genetic testing laboratory or other appropriate genetic testing through the Inborn Errors Working Party of the European Society for Blood and Marrow Transplantation. Centers in Europe, Australia, Canada, the USA, and the Middle East were contacted. Of 50 centers approached, 25 responded, and 16 contributed patients. Detailed patient information was collected via structured questionnaires. A total of 61 patients from 13 countries met inclusion criteria and were classified by disease phenotype at presentation (HMIA vs. IBD) according to the treating physician.

Questionnaires were reviewed between January and December 2024, and data were entered into a dedicated database. Demographic, clinical, immunological, and treatment-related information was collected. Outcomes included survival, long-term complications (secondary tumors, autoimmunity), immunological status, and quality of life at the last follow-up.

### Variables

The primary outcome was to describe the clinical characteristics of children with TTC7A deficiency. Variables recorded were baseline characteristics: age at symptom presentation, age at genetic diagnosis, sex, *TTC7A* genotype, gastrointestinal phenotype (HMIA or IBD), liver derangement at diagnosis, need for surgery at diagnosis, need for PN and age at initiation, indication for PN (defined as secondary to surgery/transplant, due to partial enteral tolerance, or no enteral tolerance).

Moreover, immunological and infectious characteristics were recorded: newborn screening diagnosis, CD3/CD19/CD56 counts at diagnosis, presence of hypogammaglobulinemia and cytopenia, infections at diagnosis (fungal/viral/bacterial), use of antibacterial/antifungal prophylaxis, use of immunoglobulin, and the need for intensive care unit admission.

Data about treatment received were also recorded: need for immunosuppression, how many lines and which type, response, need for intestinal or liver transplant, and data about it. Secondary outcomes were collected for those who had undergone HSCT and included age at HSCT, type of HSCT (matched/mismatched related/unrelated), conditioning regimen (RIC/MAC), presence of aGVHD or cGVHD, and incidence of rejection.

Outcomes at the last follow-up recorded were as follows: status (alive/dead), cause of death (transplant-related mortality, infections, malignancy, immune dysregulation, disease progression), age at the last follow-up, presence of autoimmune manifestations or malignancy, CD3 counts at the last follow-up, need for immunosuppression, or immunoglobulin replacement.

### Statistical analysis

Descriptive statistics were applied, with all variables treated both as categorical and as continuous. Continuous variables were assessed for normality and presented as means with standard deviation or median with interquartile range, as appropriate. Categorical variables were presented as frequencies (%). The Fisher exact test was used to compare characteristics, treatments, complications, and outcomes between HMIA and IBD groups. Overall survival was estimated using the Kaplan–Meier method with 95% confidence intervals. Eureka Statistics and Excel were used for the statistical analysis. The difference in survival distributions between groups was assessed using the log-rank test.

## Ethical considerations

The study was conducted in accordance with the Declaration of Helsinki and Good Clinical Practice, with approval from local institutional review boards or ethics committees in accordance with local policies for retrospective data reporting (15, 16, 17).

## Data Availability

The data are available from the corresponding author upon reasonable request.
